# Rare variant density across the genome and across populations

**DOI:** 10.1186/1753-6561-5-S9-S39

**Published:** 2011-11-29

**Authors:** Paola Raska, Xiaofeng Zhu

**Affiliations:** 1 Department of Epidemiology and Biostatistics, Case Western Reserve University, 10900 Euclid Ave., Cleveland, OH 44106, USA

## Abstract

Next-generation sequencing allows for a new focus on rare variant density for conducting analyses of association to disease and for narrowing down the genomic regions that show evidence of functionality. In this study we use the 1000 Genomes Project pilot data as distributed by Genetic Analysis Workshop 17 to compare rare variant densities across seven populations. We made the comparisons using regressions of rare variants on total variant counts per gene for each population and Tajima’s *D* values calculated for each gene in each population, using data on 3,205 genes. We found that the populations clustered by continent for both the regression slopes and Tajima’s *D* values, with the African populations (Yoruba and Luhya) showing the highest density of rare variants, followed by the Asian populations (Han and Denver Chinese followed by the Japanese) and the European populations (CEPH [European-descent] and Tuscan) with the lowest densities. These significant differences in rare variant densities across populations seem to translate to measures of the rare variant density more commonly used in rare variant association analyses, suggesting the need to adjust for ancestry in such analyses. The selection signal was high for *AHNAK*, *HLA-A*, *RANBP2*, and *RGPD4*, among others. *RANBP2* and *RGPD4* showed a marked difference in rare variant density and potential selection between the Luhya and the other populations. This may suggest that differences between populations should be considered when delimiting genomic regions according to functionality and that these differences can create potential for disease heterogeneity.

## Background

Genome-wide association studies have proven to be successful in identifying some of the common variants that contribute to complex disease. Nevertheless, most of the variation underlying complex disease has yet to be discovered. Focus is starting to shift toward capturing other types of variation. The new next-generation sequencing technology allows for the identification of rare variants across many individuals and for their use in studies of association to disease. This new technology also allows for the study of patterns of sequence variation that can point to functional regions in the genome, which can then be targeted in candidate gene and pathway association analyses. Incorporating a priori knowledge about functionality across genomic regions lowers the number of association tests conducted in the usual genome-wide association studies, thereby increasing their power.

Rare variant association analysis can be conducted under the premise that rare variants are deleterious and that their presence in individuals increases risk of disease. Several approaches to comparing counts of rare variants between case and control subjects have been proposed, including individual- and group-based measures of diversity [1,2]. One such measure could be the number of rare variants for the group relative to the total number of polymorphic sites. Differences between populations in the genome-wide density of rare variants can confound rare variant association analysis, and such differences between populations have yet to be described.

Rare variant density can also be used to narrow down the functional regions within the genome. Neutrality theory suggests that most mutations are selectively neutral; that is, the allele frequency distribution observed for a population is the result of a balance between the acquisition of new mutations and the loss of mutations as a result of genetic drift [3]. Tajima’s *D* is one common measure used to gauge excess or deficiency of rare variants under the null model of neutral evolution [4]. Populations that differ in their environmental histories and that present some genome-wide divergence (and therefore different genomic contexts) can vary with respect to the regions or genes that undergo selection [5]. These differences may be relevant for studies that attempt to incorporate regional genomic functionality into the study’s design.

In this paper we look at the patterns of rare variant density across the genome using the sequencing data obtained by the 1000 Genomes Project [6] and distributed by Genetic Analysis Workshop 17 (GAW17) for 3,205 genes across seven populations: CEPH (a European-descent population from Utah) (*n* = 90), Denver Chinese (*n* = 107), Han Chinese (*n* = 109), Japanese (*n* = 105), Luhya (*n* = 108), Tuscan (*n* = 66), and Yoruba (*n* = 112). We examine differences in rare variant distribution between populations and discuss their implications for rare variant association analysis and for inferring genome region functionality.

## Methods

### Rare to total variant ratios across genes and populations

Rare variant counts (single-nucleotide polymorphisms [SNPs] with minor allele frequencies [MAFs] less than 0.05) and total variant counts (total number of SNPs) are calculated for each of the 3,205 genes (*i* = 1:3,205) for each population individually and for the seven populations pooled (*j* = 1:8). A linear regression between these two measures gives an estimate for the slope *B*, which characterizes their relationship:

Rare variants (*i*, *j*) = *B*(*j*) × Total variants (*i*, *j*) + *e*(*i*, *j*). (1)

Differences between population slopes are tested for significance using the Student’s *t*-test statistic, computed as the difference between the slope estimates (*B*) divided by the standard error (SE) of this difference:(1)

The same analysis is carried out using synonymous and nonsynonymous SNPs separately.

### Individual-based rare variant density measures

The Yoruba and CEPH populations are contrasted with respect to their rare variant density across individuals. The proportion of individuals with rare variants is computed for each of the 3,205 genes as the number of individuals who have at least one rare variant for the gene for their population divided by the total number of individuals in the population. The average number of rare variants per individual for each of the genes is computed by summing the number of rare variants present in each individual for the gene and dividing by the total number of individuals in the population. A paired *t* test is used to compare the two populations.

### Tajima’s *D*

Two measures of nucleotide diversity are *θ* and *π*. *θ* is the total number of segregating sites and is given by:(2)

where *S* is the number of segregating sites in gene and *n* is the population size. *π* is based on the levels of heterozygosity at the individual sites and is given by:(3)

These two measures are contrasted in Tajima’s *D* value:(4)

The variance of the difference between these two measures is computed as described by Tajima [4].

Our analysis was made using nonsynonymous and synonymous SNPs separately. All computations were carried out through programming with R software using its standard base packages.

## Results

**Figure 1:**
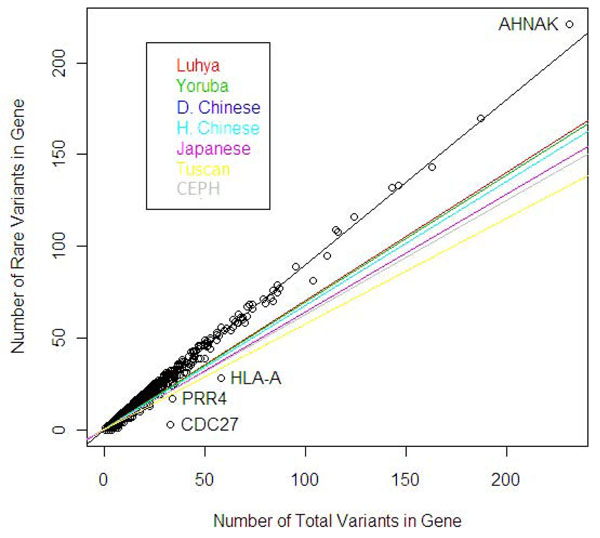
**Rare variant density for each population and all populations pooled using all available SNPs**. The slope for the linear regression of rare variants on total variants per gene for all populations pooled is shown in black (each point represents an individual gene). The highlighted genes are those with the greatest difference between observed and predicted values. The slopes for the seven individual populations are shown in different colors. The Denver Chinese (D. Chinese) and Han Chinese (H. Chinese) are superimposed in such a way that only one line is observed for both slopes.

We found significant differences for the slopes describing the relationship between rare variants and total variants per gene across populations in the analysis using all SNPs (Figure 1; Table 1). The only exceptions are the comparison between the Han and the Denver Chinese populations and that between the Luhya and Yoruba populations, which do not present significant differences. Because these differences in slope reflect the overall rare variant density throughout the genome, demographic differences between the populations rather than selection differences are likely responsible for the result. The Luhya and Yoruba populations have the highest slopes, representing a higher load of rare variants relative to the number of polymorphisms, whereas the Tuscan and CEPH populations present the lowest slopes. When all the populations are pooled, the slope becomes significantly higher.

**Table 1:** Rare variant density and values of Tajima’s *D* across populations

Population	Slope			Mean Tajima’s *D*	
	
	Estimate	Standard Error	*p*-value	Nonsynonymous SNPs	Synonymous SNPs
Tuscan	0.576	0.005	3.30 × 10^−15 a^	−0.097	0.161
CEPH	0.626	0.004	5.90 × 10^−3 a^	−0.151	0.140
Japanese	0.644	0.005	6.60 × 10^−8 a^	−0.197	0.090
Han Chinese	0.677	0.004	9.33 × 10^−1^	−0.296	−0.032
Denver Chinese	0.677	0.004	1.30 × 10^−3 a^	−0.265	−0.028
Yoruba	0.694	0.003	8.80 × 10^−2^	−0.461	−0.213
Luhya	0.701	0.003	<2.2 × 10^−16 a^	−0.444	−0.215
All	0.902	0.002			

Rare variant to total variant slope estimates and standard errors are given for each population and for all the populations pooled. The populations are ranked according to slope. Equality of slopes for contiguous populations (i.e., Tuscan vs. CEPH, CEPH vs. Japanese, etc.) is tested, and the *p*-value for each comparison is given. Finally, the mean values of Tajima’s *D* for each population calculated on the basis of nonsynonymous and synonymous SNPs are given.

^a^ Significant difference between the population and the one immediately below.

For the pooled analysis, we found that the outlying genes were *AHNAK*, which had the greatest number of rare variants per total number of variants compared to other genes, and *CDC27*, *PRR4*, and *HLA-A*, which had the least number of rare variants per total number of variants.

The rare variant to total variant slopes and Tajima’s *D* values for the nonsynonymous and synonymous SNPs group the populations in a similar way (see Table 1). The Tuscan and CEPH populations present the lowest density of rare variants, with their lower slope values and higher *D* means, followed by the Japanese population. The Chinese populations present an intermediate value, and the African Yoruba and Luhya populations have the highest slopes and most negative *D* values.

**Figure 2:**
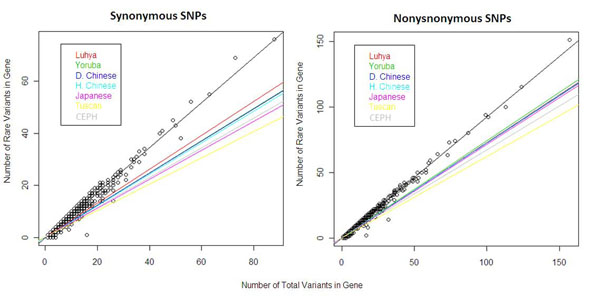
**Comparison of rare variant density when using synonymous and nonsynonymous SNPs**. Synonymous SNPs show greater differentiation between the populations. D. Chinese, Denver Chinese; H. Chinese, Han Chinese.

For the separate analyses on synonymous and nonsynonymous SNPs, we obtained larger differences between population slopes when we used the synonymous SNPs (Figure 2). The analysis with synonymous SNPs presents a similar pattern and similar statistical significance to the analysis using all SNPs. For the analysis using only nonsynonymous SNPs, we found that only the Tuscan and CEPH populations had significantly different slopes compared to each other and to the rest of the populations.

**Figure 3:**
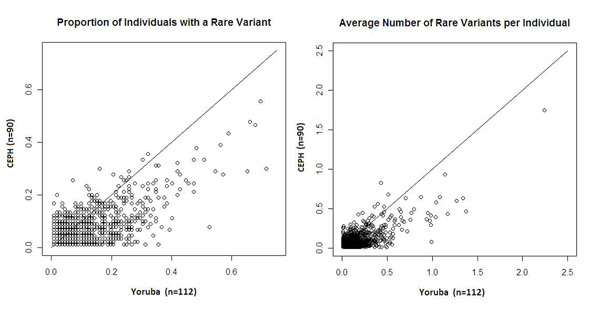
**Measures of rare variant density used in rare variant case-control association analysis**. Comparison of the distribution of rare variants between the Yoruba and CEPH populations. Each point represents the value for one of the genes for both populations. The identity lines are plotted to aid in the comparison.

As with the group-based rare variant density measure represented in the rare variant to total variant regressions, the individual-based rare variant density measures show a greater rare variant density in the Yoruba population than in the CEPH population (Figure 3). The mean proportion of individuals with a rare variant across all genes is 0.101 for the Yoruba population and 0.072 for the CEPH population (*p* < 2.2 × 10^−16^). For the average number of rare variants per individual the values are 0.075 and 0.055, respectively (*p* < 2.2 × 10^−16^).

**Figure 4:**
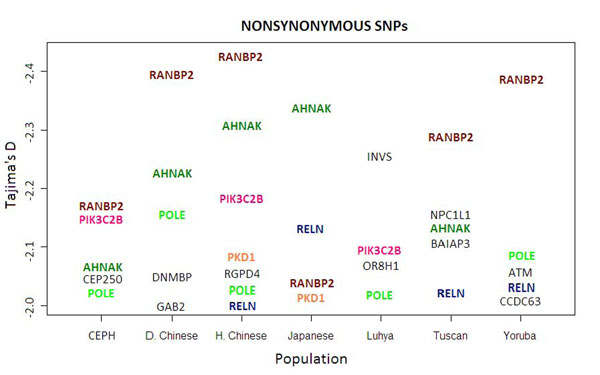
**Values of Tajima’s *D* less than −2 using nonsynonymous SNPs for all populations.** Genes present in more than one population are presented in color. D. Chinese, Denver Chinese; H. Chinese, Han Chinese.

**Figure 5:**
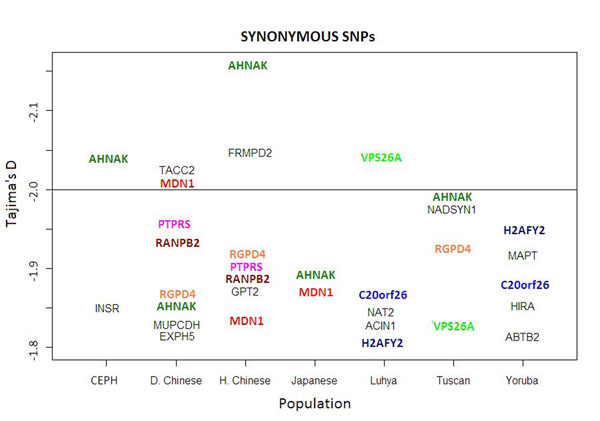
**Values of Tajima’s *D* less than −1.8 using synonymous SNPs for all populations.** Genes present in more than one population are presented in color. D. Chinese, Denver Chinese; H. Chinese, Han Chinese.

In the analysis using only nonsynonymous SNPs, the mean value of Tajima’s *D* for each population (see Table 1) is more negative than when only synonymous SNPs are used. In addition, in the nonsynonymous SNP analysis, there are more genes with values of Tajima’s *D* less than −2, the lower end of the theoretical 95% confidence interval for this measure (compare Figures 4 and 5). Also, the nonsynonymous SNPs analysis shows more consistency: The genes show highly negative values of Tajima’s *D* across populations; that is, the genes show an excess of rare variants. This can be seen in the lower variance of the values of Tajima’s *D* across populations that is obtained through nonsynonymous SNPs (average = 0.18) compared to the variance observed with the synonymous SNPs (average = 0.59) (see Table 2).

**Table 2:** Statistics aggregated across populations

Gene	Mean Tajima’s *D*	Variance of Tajima’s *D*	Total number of variants	Proportion of rare variants	Proportion of rare variants that are nonsynonymous
**Nonsynonymous**					
*AHNAK*	−2.08	0.06	65.86	0.86	0.69
*RELN*	−1.97	0.02	33.43	0.74	0.59
*POLE*	−1.95	0.04	24.29	0.72	0.69
*PIK3C2B*	−1.83	0.19	21.29	0.62	0.56
*PKD1*	−1.49	0.39	22.86	0.71	0.61
*RANBP2*	NA	NA	24.57	0.82	NA
*INVS*	−1.72	0.22	9.43	0.82	0.75
*NPC1L1*	−1.70	0.10	13.71	0.83	0.58
*BAIAP3*	−1.73	0.05	13.29	0.81	0.63
*RGPD4*	−1.16	0.83	18.86	0.83	0.62
*CEP250*	−1.59	0.06	17.14	0.85	0.65
*ATM*	−1.38	0.19	5.29	0.95	0.77
*GAB2*	NA	NA	9.14	0.54	NA
*DNMBP*	−1.82	0.04	19.43	0.69	0.66
*OR8H1*	−1.24	0.29	6.86	0.63	0.93
*CCDC63*	−1.64	0.04	8.00	0.90	0.77
Average	−1.66	0.18	19.59	0.77	0.68

**Synonymous**					
*TACC2*	−1.20	0.36	36.71	0.67	0.63
*FRMPD2*	−1.21	0.48	14.14	0.78	0.47
*VPS26A*	−1.33	0.36	0.57	NA	NA
*MDN1*	−1.20	0.95	54.29	0.71	0.64
*PTPRS*	−1.21	0.36	18.86	0.78	0.37
*RGPD4*	−1.62	0.13	18.86	0.83	0.62
*C20ORF26*	−0.89	1.48	21.71	0.73	0.71
*H2AFY2*	NA	NA	3.29	NA	NA
Average	−1.24	0.59	21.05	0.75	0.57

Tajima’s *D*, its variance, total number of polymorphisms, and the proportions of rare and nonsynonymous variants are given for each of the genes that reflect the most negative values of Tajima’s *D* (less than −2 for the analysis based on nonsynonymous SNPs and less than −1.8 for the analysis based on synonymous SNPs). NA (not applicable) corresponds to instances in which the value is not defined for at least one of the populations.

*RANBP2* stands out as an exception to the consistency in values of Tajima’s *D* across populations in the nonsynonymous SNPs analysis. Excluding the Luhya population, the average number of total variants across the remaining populations is 28.5. Ninety-five percent of these variants are rare, and 75% of these rare variants are nonsynonymous. The Luhya population, on the other hand, has only one common variant for *RANBP2*. A similar pattern is observed for *RGPD4*, where the mean number of total variants is 21.5 excluding the Luhya. Eighty-five percent of these are rare, and 64% of these rare variants are nonsynonymous. The Luhya population has only three segregating sites for *RGPD4*, two of which are rare variants.

## Discussion

When comparing rare variant density between populations, investigators want to know whether the differences are due to drift or selection. For instance, values of Tajima’s *D* that are less than −2 may indicate positive selection, whereas values greater than 2 may provide evidence of balancing selection. But, under neutral theory, selection can be inferred from Tajima’s *D* only if a balance between the mutation rate and the rate of allelic fixation is assumed. This balance does not exist if the population is going through a period of expansion or contraction—demographic phenomena that can also result in negative and positive values of Tajima’s *D*, respectively—instead of selection. One way to distinguish between demography and selection is that demography affects the genome as a whole, whereas selection has a differential effect on functional regions within the genome.

Rare variant to total variant regression across the 3,205 genes distributed throughout the genome averages out the differential effects of selection. Slope differences between populations are a better reflection of the differences in demographic phenomena that affect all genes for each population. Populations group by continent for this parameter, with the African, European, and Asian populations showing slopes with more similar values within continents. The Japanese population is significantly different from both the European and the Chinese populations. Mean values of Tajima’s *D* for each population for both synonymous and nonsynonymous SNPs group the populations in the same manner, with the African populations showing the greatest density of rare variants. Note that this does not simply indicate higher and lower levels of overall variation but rather higher and lower levels of rare variants given the amount of total variation. Although the African populations have been known to harbor greater overall variation, they may also present greater levels of rare variants, even after taking their overall level of variation into account. In a study of one of the most sequenced genes in humans, *BRCA1*, individuals with African ancestry were shown to have a significantly higher proportion of deleterious mutations compared to European Americans [7]. It would be of interest to see whether this is a result that is specific to this particular gene or whether it is product of the demography of the African population, as suggested by this study, and thereby reflected genome-wide. With the advent of more affordable sequencing technologies, the answer to this question can be pursued and may aid in the study of the genetic epidemiology of cancer and health disparities.

The outlier genes highlighted in Figure 1 are likely to represent instances of selection because their rare variant loads are distinct from that of other genes and is unlikely to be due to demographic parameters. *AHNAK*, which lies above the regression line, presents an excess of rare variants compared to other genes and is likely under positive selection; *CDC27*, *PRR4*, and *HLA-A* are under the regression line, which means that they present an excess of common variants and are likely under balancing selection. The *AHNAK* gene encodes an unusually large protein that is typically repressed in cell lines derived from human neuroblastomas and in several other types of tumors. *AHNAK* is known to be composed of highly conserved repeated elements [8]. Regions in the genome that are highly conserved are generally so because of the selective pressure acting on them. At the other end of the spectrum lies the human leukocyte antigen system (*HLA*), the major histocompatibility complex in humans. *HLA* loci are known to be exceptionally diverse. As Apanius et al. [9] describe, only natural selection for heterozygosity can account for such a level of diversity.

Although the number of rare variants in relation to the number of total variants or segregating sites is not a measure that is generally used for case-control rare variant association analysis, Bansal et al. [1] pointed out that summary group-level statistics such as this can potentially be used along with individual-based measures. As they pointed out, the use and power of such statistics have not yet been assessed [1]. One possible drawback of using group diversity measures in an association test is that population substructure within the group can inflate the statistic, as can be seen by the higher slope obtained for the pooled populations (see Figure 1). Controlling for this level of substructure would have to be considered in any method that relies on such measures.

The group-level rare to total variant ratio, which is the rare variant density measure investigated here for each population, can translate into differences in number of individuals who carry a rare variant and/or number of rare variants per individual, measures more commonly used in association analyses on rare variants [2]. Two assumptions would have to hold for this translation: (1) The rare variant distribution across individuals does not differ between populations, and (2) the allele frequency spectrum for the rare SNPs does not differ between populations. Without testing these assumptions, we do see that the difference between the Yoruba and CEPH populations is also present for these measures (see Figure 3). Ancestry can therefore be an important confounder for rare variant association analysis in the same way it has proven to be for common SNP associations in genome-wide association studies. Simulations that study the best way to control for ancestry for rare variant association analysis still have to be performed. Admixed populations in particular may require a local level of ancestry control in order to account for differential rare variant densities throughout the genome.

This set of data does not afford much power for detecting significant values of Tajima’s *D* for individual genes, especially after accounting for multiple comparisons. Despite this, carrying out separate analyses on synonymous and nonsynonymous SNPs provides a way of seeing a result that can best be explained by selection. If random noise explained the rare variant distribution across the genome seen in this data set, then synonymous SNPs would necessarily present the same allele frequency spectrum as the nonsynonymous SNPs. Values of Tajima’s *D* for both sets of SNPs would not systematically differ. Here we see that they do; the nonsynonymous SNPs result in more negative values of Tajima’s *D* for all the populations (see Table 2). This may indicate an increase in power for detecting instances of selection across the genome when using the nonsynonymous SNPs, which are more likely to have a functional effect relative to the synonymous SNPs. If overall there are more instances of positive selection (negative Tajima’s *D*) than of balancing selection (positive Tajima’s *D*), then the increase in power would result in the more negative mean value of Tajima’s *D* for the population. This result is therefore a good indication that the nonsynonymous SNP analysis of Tajima’s *D* is picking up real signals of selection that are present in these data.

The contrast between the synonymous and nonsynonymous SNPs rare to total variant regressions (see Figure 2) also serves to distinguish whether selection or drift is accounting for the results. The observed population slope differences are likely caused by phenomena that affect the genome as a whole, such as genetic drift. It is to be expected, therefore, that because synonymous SNPs are less sensitive to functional variation across the genome, they can provide greater power for detecting these genome-wide differences caused by drift.

In this study we focus on the results for positive selection (excess of rare variants, negative values of Tajima’s *D*) rather than for balancing selection (excess of common variants, positive values of Tajima’s *D*) because of the ascertainment bias that is present in the data. The SNP discovery process presents a bias toward common SNPs that has not been corrected for [10], making any inference on positive values of Tajima’s *D* extremely difficult. Also, positive selection is likely to play a larger role across the genome than balancing selection would. Despite this, the rare variant to total variant regressions present an alternative to biased values of Tajima’s *D* for inferring balancing selection. The outliers under the regression line clearly show an excess of common variants that cannot be explained by ascertainment bias.

Finally, values of Tajima’s *D* provided by the nonsynonymous SNP analysis should provide relatively low variation across populations if this analysis is indeed improving the detection of functionally important genes. It is a biology that is likely to translate across different populations, and therefore the same genes should present the more extreme values from population to population. For the synonymous SNPs, on the other hand, genes are more likely to present extreme values by chance alone, and more variation across populations should be expected. This is in fact what we see here, and it serves as another indication that the nonsynonymous SNP analysis is picking up on real selection signal rather than random noise.

If this is the case, we can use our analysis to compare selection signals and what may be functionality across populations. Table 2 shows that most genes that may be presenting a selection signal have consistently highly negative values of Tajima’s *D* across all populations. One exception to this rule is *RANBP2*. *RANBP2*, like *AHNAK*, is a highly conserved gene. Its insufficiency has been linked to autosomal dominant necrotizing encephalopathy, among other things [11]. Not only is there evidence for selection for the gene in itself, but the gene has also been the object of extensive duplication in the human lineage, with the resulting region occupying approximately 10% of chromosome 2 [12]. There are eight partial copies of *RANBP2* within this region. This gene family has been named *RGP* (RanBP2-like, GRIP domain containing proteins) [12]. Evidence suggests that all eight copies are evolutionarily active and are expressed [11]. This includes *RGPD4*, the other gene in this study that showed the same pattern as *RANBP2*.

Several possibilities can account for the striking difference between the rare variant load for the Luhya population compared to the rest of the populations for these RGPD4 and *RANBP2*. Environmental differences can certainly account for differences in selection pressure between populations even when the biology and functionality of the gene remains unchanged from population to population. On the other hand, the gene’s functionality itself may be different. Genomic context, which plays a role in the functionality of individual genes, can be greatly divergent between populations because of drift and other historical events, such as deletions and duplications. This result points out a specific instance in which it may be important to take into account the ancestral composition of the study population when attempting to narrow down genomic searches according to functionality. It also underlines the potential for disease heterogeneity across different populations.

## Conclusions

Rare variant density differences exist between populations because of differences in their demographic histories and selection pressures. These differences have implications for conducting rare variant association analyses and for studies that attempt to use functionality as a means of delimiting the genomic search space for associations. They also bring to light the potential for disease heterogeneity across different populations.

## Competing interests

The author(s) declare that there is/are no competing interest(s).

## Authors’ contributions

PR conceived of the study and drafted the manuscript. XZ participated in its design and helped to draft the manuscript. All authors read and approved the final manuscript.
